# Poly(Lactic Acid)/ZnO Bionanocomposite Films with Positively Charged ZnO as Potential Antimicrobial Food Packaging Materials

**DOI:** 10.3390/polym11091427

**Published:** 2019-08-30

**Authors:** Insoo Kim, Karthika Viswanathan, Gopinath Kasi, Kambiz Sadeghi, Sarinthip Thanakkasaranee, Jongchul Seo

**Affiliations:** Department of Packaging, Yonsei University, 1 Yonseidae-gil, Wonju-si, Gangwon-do 26493, Korea

**Keywords:** bionanocomposite, poly(lactic acid), zinc oxide, antibacterial activity, interfacial interaction

## Abstract

A series of PLA/ZnO bionanocomposite films were prepared by introducing positively surface charged zinc oxide nanoparticles (ZnO NPs) into biodegradable poly(lactic acid) (PLA) by the solvent casting method, and their physical properties and antibacterial activities were evaluated. The physical properties and antibacterial efficiencies of the bionanocomposite films were strongly dependent on the ZnO NPs content. The bionanocomposite films with over 3% ZnO NPs exhibited a rough surface, poor dispersion, hard agglomerates, and voids, leading to a reduction in the crystallinity and morphological defects. With the increasing ZnO NPs content, the thermal stability and barrier properties of the PLA/ZnO bionanocomposite films were decreased while their hydrophobicity increased. The bionanocomposite films showed appreciable antimicrobial activity against *Staphylococcus aureus* and *Escherichia coli*. Especially, the films with over 3% of ZnO NPs exhibited a complete growth inhibition of E. coli. The strong interactions between the positively charged surface ZnO NPs and negatively charged surface of the bacterial membrane led to the production of reactive oxygen species (ROS) and eventually bacterial cell death. Consequently, these PLA/ZnO bionanocomposite films can potentially be used as a food packaging material with excellent UV protective and antibacterial properties.

## 1. Introduction

Food packaging materials play an important role in protecting the food product from environmental factors such as moisture, light, oxygen, microbes, mechanical stress, and dust. Therefore, such materials must exhibit a higher barrier property to retard the oxygen or water permeability, the strong antimicrobial activity toward bacteria or mold, high mechanical strength to environmental stress, and good UV-protective property in order to protect the food, maintain quality, and prolong the shelf life of food products [1]. In recent years, consumer awareness about packaging disposal and price hikes of petro-based packaging materials has increased and this has motivated research efforts toward developing bio-based and biodegradable polymers [2]. There has been an augment in the demand for environment-friendly and non-toxic packaging in the market. For such needs, poly(lactic acid) (PLA) is one of the best alternatives to replace petrochemical-based commodity plastics, as it can reduce the carbon footprint and waste disposal threats to the environment. PLA is a biodegradable polymer, which is obtained by the bacterial fermentation of bio-resources, such as sugar beet or corn starch. Additionally, PLA has received the Generally Recognized as Safe (GRAS) tag from the United States Food and Drug Administration, and its feasibility in the beverage packaging application is approved [3,4,5].

PLA has also been considered as a potentially useful material in fields such as tissue engineering, drug delivery, and dentistry [6]. PLA exists as three different crystal types, such as *α*, *β*, and *γ*. The *α*-form crystal of PLA is the most common type, which has an orthorhombic crystalline shape with a 103 helical chain conformation. PLA is commonly prepared via the melting or solution methods, and has been described as a chiral polymer containing asymmetric carbon atoms with a helical conformation. The three kinds of crystallinity, such as: (i) Amorphous, (ii) partial crystalline, and (iii) nearly half-crystalline, are related to the l-lactide present in the PLA backbone at 87.5, 92, and 100%, respectively. Depending on the l-Lactide in the PLA backbone, they show the melting point of 150–180 °C and the glass transition temperature of 50–60 °C, respectively [7,8,9]. PLA has been considered as an advanced packaging material because of its biodegradability, and good mechanical and physicochemical properties [7]. However, PLA has some drawbacks such as brittleness, a weak barrier property, poor UV-blocking ability, low heat distortion temperature, and low melt viscosity. Therefore, these properties need to be improved before application in food packaging. Nevertheless, it must be noted that such properties are not sufficient to maintain the quality and extend the shelf-life of a food product, and therefore, bio-based packaging materials with antimicrobial properties need to be developed. 

The introduction of nanofillers, such as organo-modified layered silicates, silver, zinc oxide, graphite derivatives, carbon nanotubes, and silica, is a practical and feasible method to enhance the antibacterial, barrier, thermal, and mechanical properties of PLA [4,10,11]. Although silver and some metal-based nanoparticles (NPs) are known to display strong antibacterial properties, their cost limits wider applications. On the other hand, some metal oxide NPs, such as ZnO and TiO_2_, are commonly used for health-oriented applications because of the cost-effectiveness, convenience, and unique physicochemical characteristics [12]. The ZnO NPs have also been used as multifunctional inorganic fillers because of their environmental compatibility. The ZnO NPs are also used in solar cell fabrication as photocatalysts in the pharmaceutical, cosmetic, and food packaging industries [13]. The ZnO is a direct wide-band-gap semiconductor with a band gap of 3.37 eV. In addition, it has been reported that ZnO NPs with positively charged surface exhibit stronger antimicrobial activities against Gram-negative bacteria than Gram-positive bacteria. Compared to Gram-positive bacteria, Gram-negative bacteria have additional negative charges on their surfaces and consequently, they tend to bond strongly with the positively charged surface materials. The presence of a thicker peptidoglycan layer in the Gram-positive bacteria makes them less sensitive to ZnO NPs in comparison to Gram-negative bacteria [14]. The higher surface-to-volume ratio can exponentially enhance the surface interactions, which results in bacterial cell death [15]. Introducing NPs into the polymer matrix enables a direct contact of NPs with the bacterial cell, leading to cell structural change and damage. Several researchers have studied the antimicrobial activity of ZnO NPs incorporated into the polymer matrix [11,12,15].

In our previous study, the authors synthesized ZnO NPs using the didodecyldimethylammonium bromide (DDAB) surfactant and investigated their antimicrobial activity [16]. The ZnO NPs synthesized by ionic liquid DDAB had a positive surface charge, whereas those synthesized without DDAB had a negative surface charge. The ZnO NPs with positive surface charge showed remarkably stronger antimicrobial activity against Gram-positive and Gram-negative bacteria as compared to ZnO NPs with a negative surface charge. Arakha et al. (2015) reported that the positively surface charged ZnO NPs (+12.9 mV) showed better antibacterial activity compared to the negatively surface charged ZnO NPs (−12.9 mV). It is related that the positively charged ZnO NPs can easily bind with the negatively charged bacterial cell, resulting in the enhancement of ROS production and the inhibition of bacterial cell viability [17,18]. Furthermore, it is expected that negatively charged oxygen with unpaired electrons in PLA polymer would have higher interfacial electrostatic interactions with the positively charged surface ZnO NPs [11], resulting in a good dispersion in the PLA matrix and better physical properties in their bionanocomposite films [10].

In the present work, biodegradable PLA/ZnO bionanocomposite films with various contents of positively charged surface ZnO NPs were fabricated by solvent casting. To evaluate the feasibility of the PLA/ZnO bionanocomposite films as antimicrobial food packaging materials, their morphological, thermal, barrier, and antimicrobial properties were evaluated in detail. 

## 2. Experimental Section

### 2.1. Materials

Zinc nitrate hexahydrate (Zn(NO_3_)_2_·6H_2_O) and didodecyldimethylammonium bromide ([CH_3_(CH_2_)11]_2_N(CH_3_)2(Br)) (DDAB) were purchased from Sigma–Aldrich Co. (Seoul, South Korea). Ethylene glycol (99.5%), chloroform, ammonia solution (NH_3_, 28%), and ethanol (99%) were obtained from Wako Pure Chemical Co., Osaka, Japan. The Poly (l-lactic acid) (PLA) (*M*_w_ = 280 kDa, *M*_w_/*M*_n_ = 1.98) was purchased from Nature Works LLC (Lincoln, NE., USA). Double-distilled water was used in all the experiments. 

### 2.2. Synthesis of ZnO NPs

The ZnO NPs were prepared via a solvothermal method as follows [19]. First, 0.1 M Zn(NO_3_)_2_·6H_2_O and 0.005 M DDAB were dissolved in 100 mL of ethylene glycol. The mixture was stirred magnetically for 30 min, followed by the addition of 5 mL of NH_3_. Next, the reaction mixture was carefully transferred to a sealed 500-mL Teflon-lined stainless-steel autoclave and heated solvothermally at 150 °C for 24 h. The collected product was washed three times with ethanol and water before drying in a vacuum oven at 100 °C for 24 h. The dried ZnO was ground and stored at room temperature in a glass vial for further analysis. The zeta potential of the as-prepared ZnO NPs was 34.7 mV.

### 2.3. Preparation of Bionanocomposite Films

PLA (7 g) was dissolved in chloroform (93 g) under constant stirring at 25 °C for 6 h. To this PLA solution, various amounts of ZnO NPs (0–10 wt % based on PLA weight) were added under stirring. Each mixture was stirred further for 5 h and bar-coated onto a glass substrate using a bar-type automatic film coating apparatus (KIPAE E&T Co. Ltd., Hwasung, Korea) controlling the film thickness to (30 ± 1) μm. The coated films were dried in an oven at 60 °C for 24 h. The PLA/ZnO bionanocomposite films with ZnO NPs loadings of 0, 0.5, 1, 3, 5, and 10 wt % are referred to as PLA, PLA/ZnO (0.5%), PLA/ZnO (1%), PLA/ZnO (3%), PLA/ZnO (5%), and PLA/ZnO (10%), respectively.

### 2.4. Characterization

Fourier transform infrared (FT-IR) spectra of ZnO NPs, pure PLA, and PLA/ZnO bionanocomposite films were measured using a Spectrum 65 FT-IR spectrometer (PerkinElmer Co., Ltd., MA., Waltham, USA) in the attenuated total reflection (ATR) mode. The thunder dome was a single-reflection ATR accessory comprising a diamond/ZnSe (single reflection) crystal.

The X-ray diffraction (XRD) patterns of the ZnO NPs were collected and analyzed using a Bruker D8 Advance X-ray diffractometer (Billerica, MA., USA) with Cu Kα (λ = 1.54 Å) radiation as the source in the 2θ range of 10–80°. For the PLA/ZnO bionanocomposite films, high-resolution X-ray diffraction (HR-XRD) patterns were collected using an automated multi-purpose X-ray diffractometer (SmartLab 9 kW system, Rigaku Co., Tokyo, Japan) with Cu Kα radiation operating at 40 kV and a 200-mA electric current from 10 to 20 and 30 to 40° (2θ). 

The transparency and ultraviolet light barrier properties of PLA/ZnO bionanocomposite films were studied using UV–Visible (UV–Vis) diffuse reflectance spectroscopy (DRS) in the wavelength range of 250–800 nm with a UV 2600 spectrophotometer (Shimadzu Co., Tokyo, Japan) equipped with an integrating sphere. UV–Vis DRS measurements were performed for 30-μm-thick films in the transmission mode.

Scanning electron microscopy (SEM) images of the top and fractured surfaces of the PLA/ZnO bionanocomposite films were obtained using a Quanta FEG250 scanning electron microscope (FEI Co., OR., Hillsboro, USA) to examine the dispersion state of ZnO NPs in the PLA matrix. Before imaging, all samples were coated with a thin layer of Pt.

The thermal properties of the PLA/ZnO bionanocomposite films were studied using a Q10 differential scanning calorimeter (TA Instrument Co., DE., New Castle, USA). The samples of ~5.5 mg were heated from 0 to 230 °C at a rate of 10 °C/min in a nitrogen atmosphere. The thermal stability of the ZnO NPs and PLA/ZnO bionanocomposite films was evaluated using a TGA 4000 thermogravimetric analyzer (PerkinElmer Co., MA., Waltham, USA). The measurements were performed in the range of 30 to 800 °C at a heating rate of 10 °C/min in a nitrogen atmosphere. The mass of the samples was ~10 mg.

The oxygen transmission rates (OTRs) of the pure PLA film and PLA/ZnO bionanocomposite films were recorded using an OTR 8001 oxygen permeability tester (Systech Instruments Co., IL., Johnsburg, USA). The OTR tests were performed at 23 °C and 0% relative humidity (RH). The water-vapor transmission rates (WVTRs) were also measured using a Permatran-W 3/33 water-vapor permeation analyzer (Mocon Inc., Minnesota, Minneapolis, USA) at 38 °C, 90% RH, and 10 cm^3^/min nitrogen flow.

A Phoenix 300 contact-angle goniometer (SEO Co. Ltd., Suwon, South Korea) was used to measure the contact angles and surface free energies of the pure PLA film and PLA/ZnO bionanocomposite films. Both the deionized water and methylene iodide were used to perform the measurement on the sample at 25 °C, and an eyepiece was used for observation according to the θ/2 method. A micro syringe was used to maintain the volume of the sessile drop at 5 μL in all the cases. The average contact angles for five drops were taken, and the surface free energies were estimated using the harmonic-mean equation [20] according to the contact angles for water and methylene iodide on the PLA/ZnO bionanocomposite films.

The JIS Z 2801 standard was employed to perform antibacterial tests on the PLA/ZnO bionanocomposite films. The Gram-positive Staphylococcus aureus (*S. aureus*) and the Gram-negative *Escherichia coli* (*E. coli*) bacteria were individually grown on tryptic soy and MacConkey agar plates at 38 °C for 24 h, and a single colony was transferred into a 10 mL aliquot of nutrient broth. The flat portions of the sample composites were cut into 5 cm × 5 cm squares and then placed in Petri dishes. The film samples were sterilized with UV light, and 0.4 mL of the bacterial suspension was dropped on the films. Next, the samples were covered using sterilized polyethylene films and maintained at 37 °C. After 24 h, the treated films were washed off with a 0.9% saline solution, and a serial dilution of the *S. aureus* and *E. coli* bacterial suspensions was plated onto the tryptic soy and MacConkey agar plates, followed by incubation at 37 °C for 48 h. The bacterial colony-forming units (CFUs) were determined by calculating the value of antimicrobial activity (*R*) using the following equation:

*R* (%) = (*B* − C)/*B* × 100 (1)

where *B* and *C* are the CFUs of the viable bacterial cells on the pure PLA and PLA/ZnO bionanocomposite films, respectively.

## 3. Results and Discussion

### 3.1. FT-IR Analysis

**Figure 1:**
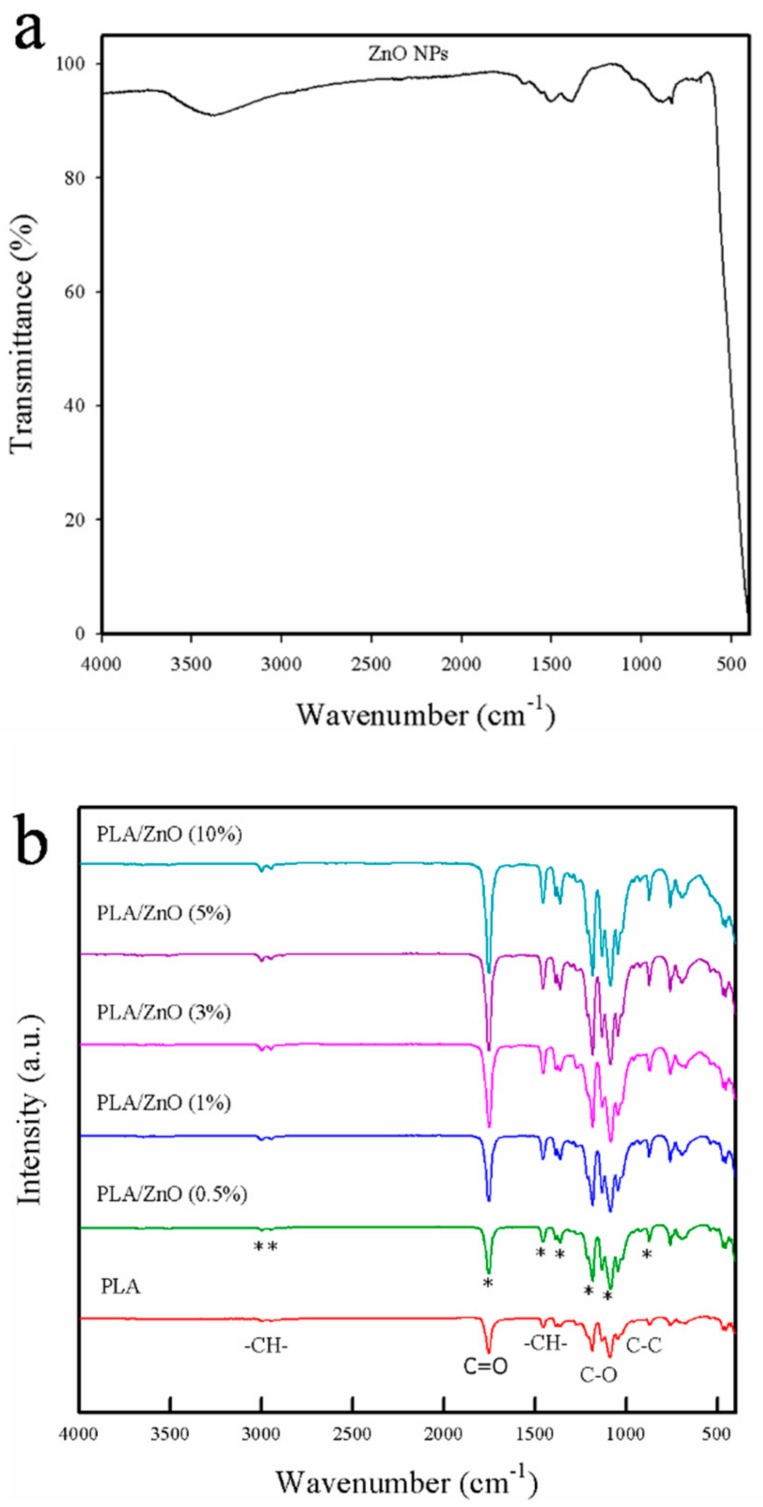
FT-IR spectra of (**a**) ZnO NPs and (**b**) pure poly(lactic acid) (PLA) film and PLA/ZnO bionanocomposite films.

Fourier-transform infrared (FT-IR) spectroscopy was employed to investigate the interfacial interactions between PLA and ZnO NPs [21]. As shown in Figure 1a, the IR spectrum of ZnO NPs showed four characteristic peaks at 3380, 1390, 882, and 684 cm^−1^ corresponding to the O–H stretching, C–C stretching, C–H bending, and weak Zn–O stretching vibrations, respectively [22]. The pure PLA film exhibited several characteristic peaks at 2948, 3001, 1749, 1454, 1370, 1267, 1174, 1080, 1090, 1045, and 870 cm^−1^, as shown in Figure 1b. The bands at ~2948 and 3001 cm^−1^ corresponded to –C–H– stretching, while the sharp absorbance band at 1749 cm^−1^ was assigned to the C=O stretching of the ester group. The absorbance bands at 1454 and 1370 cm^−1^ corresponded to the –C–H– symmetric and asymmetric deformations, respectively. The absorption bands corresponding to –C–O– stretching were also observed in the vicinity of 1267–1045 cm^−1^ [13]. The bionanocomposite films showed the same peaks as the pure PLA film. However, with the increasing ZnO NPs content, the intensities of the characteristic peaks, such as C=O stretching, –C–H– bending, and –C–O– stretching were increased because the nucleation density of PLA increased upon the introduction of ZnO NPs [3]. Compared with pure PLA, however, all PLA/ZnO bionanocomposite films showed a similar pattern without any significant shift in the peak position. This indicated that there was no appreciable chemical interfacial interaction between the PLA matrix and ZnO, which limited the extent of the improvement of their thermal and barrier properties [23,24]. 

### 3.2. XRD Analysis

**Figure 2:**
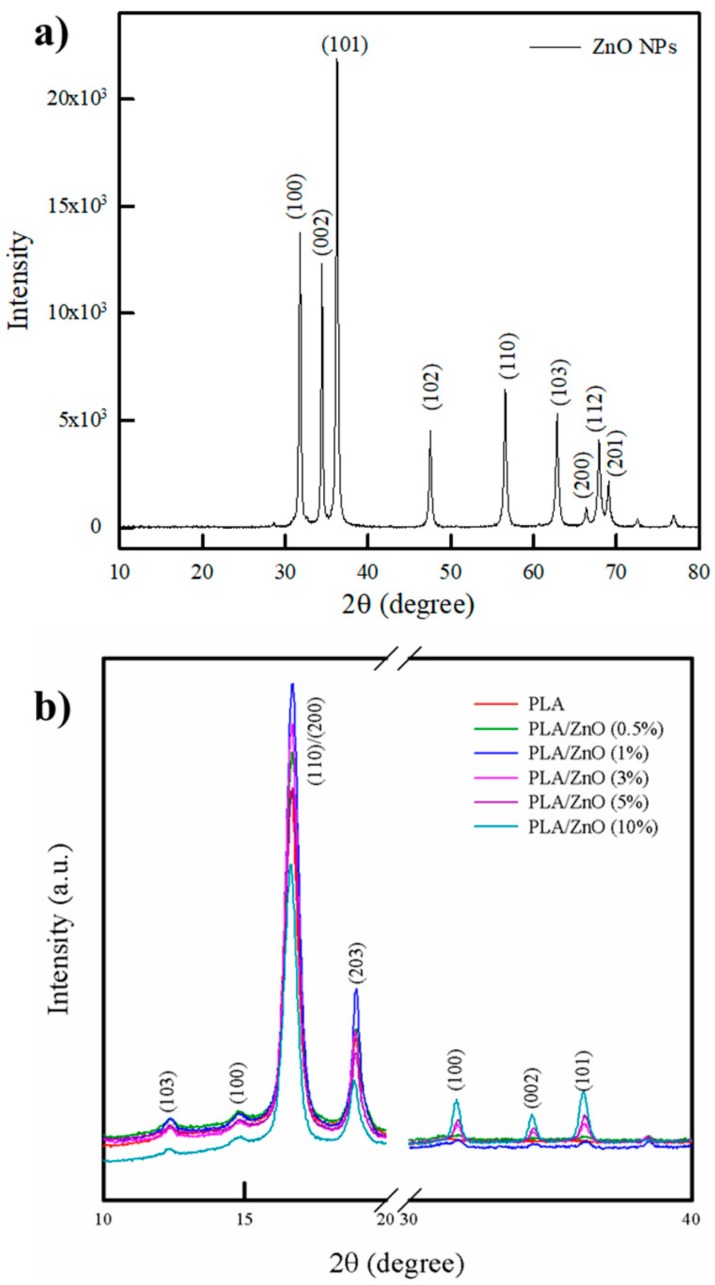
XRD patterns of (**a**) ZnO NPs and (**b**) pure PLA film and PLA/ZnO bionanocomposite films.

The XRD patterns of pure PLA and PLA/ZnO bionanocomposite films are shown in Figure 2. Generally, pure PLA has an orthorhombic crystal structure (*α*-form) and consists of 103-helical chains [25]. The pure PLA film showed characteristic peaks located at 2θ = 12.5, 14.8, 16.8, and 19.1°, which were attributed to the (103), (100), (110)/(200), and (203) planes of the *α*-form crystal, respectively [26]. Pure ZnO NPs showed characteristic peaks located at 2θ = 31.6, 34.4, 36.2, 47.4, 56.7, 62.7, 66.2, 67.9, and 69.0°, which corresponded to (100), (002), (101), (102), (110), (103), (200), (112), and (201) planes, respectively, of the hexagonal crystal of ZnO NPs (JCPDS card No 36-1451) [13]. The PLA/ZnO bionanocomposite films showed almost the same peaks as pure PLA. However, the new peaks at 2θ = 31.6, 34.2, and 36.1° corresponding to the (100), (002), and (101) planes of hexagonal wurtzite structure of ZnO were observed in the PLA/ZnO bionanocomposite films. With the increasing ZnO content, the intensity of ZnO peaks increased [13]. Generally, NPs can enhance crystallization by acting as nucleating agents, whereas they may also reduce the growth rate of spherulite by hindering ordered packing of polymer chain segments. Further, the trend depends on the system attributes such as their content, the degree of dispersion, and the extent of interfacial interactions between polymer and NPs [27]. In our study, the bionanocomposite films with relatively low content of ZnO (0.5 and 1%) showed an increase in the intensity of the PLA peaks (12.5, 14.8, 16.8, 19.1, and 22.3°), indicating an increase in crystallinity. This suggests that the small amount of ZnO NPs with relatively good dispersion acted as nucleating agents, which led to an increase in the crystallinity of PLA. However, the bionanocomposite films with over 3% ZnO NPs showed a decrease in the intensity of the PLA peaks (12.5, 14.8, 16.8, 19.1, and 22.3°), indicating a reduction in crystallinity. This implied that the agglomeration of ZnO NPs with poor dispersion led to the distribution of the intermolecular interactions between the PLA chains, resulting in a reduction of chain regularity and a decrease in crystallinity. Our result is consistent with those of other research works [24,28].

### 3.3. Optical Property

**Figure 3:**
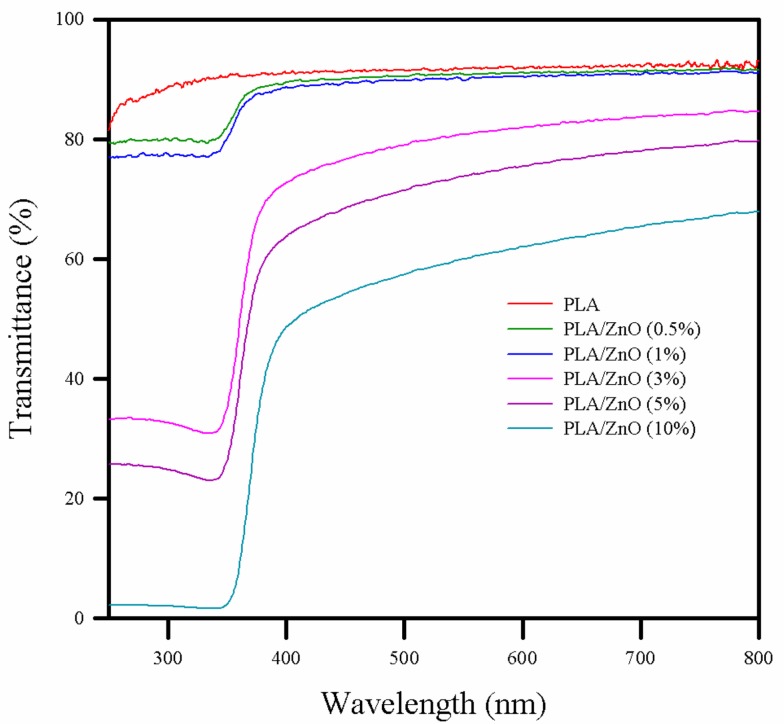
UV–Vis spectra of pure PLA and PLA/ZnO bionanocomposite films.

The transmission of ultraviolet radiation (UV) (100–400 nm) and visible light (400–700 nm) is an important parameter for packaging design in preserving and protecting products because the photochemical degradation of polymer can affect its performances, while the packaging material properties should be maintained until the food product reaches consumers. In addition, consumers prefer transparent materials to check the food produce inside the packaging. Therefore, the preparation of advanced materials to meet the transparency and the UV-light barrier requirements is worth investigating [7,29]. The UV–Vis transmittance spectra of pure PLA and PLA/ZnO bionanocomposite films are shown in Figure 3. The pure PLA film exhibited a very high transmittance in both the UV and visible ranges, indicating low UV blocking and high transparency. With the increasing ZnO NPs content, i.e., to 0.5 and 1%, the bionanocomposite films showed a slight decrease in the transmittance in the UV range, but there was no significant change in transmittance in the visible range. At a higher content of ZnO NPs (over 3%), the bionanocomposite films showed a dramatic decrease in the transmittance to ~60% in the UV range, but a mild decrease of ~20% in the visible range. This observation implied that the bionanocomposite films showed excellent UV absorption without a considerable change in transparency as compared to the pure PLA film. The observed change in the transmittance of the PLA/ZnO bionanocomposite films in the UV range is related to the process of electron excitation (band-gap absorption) in ZnO NPs [30]. Therefore, the PLA/ZnO bionanocomposite films were UV-protective and could be potentially applied in food packaging.

### 3.4. Morphology

**Figure 4:**
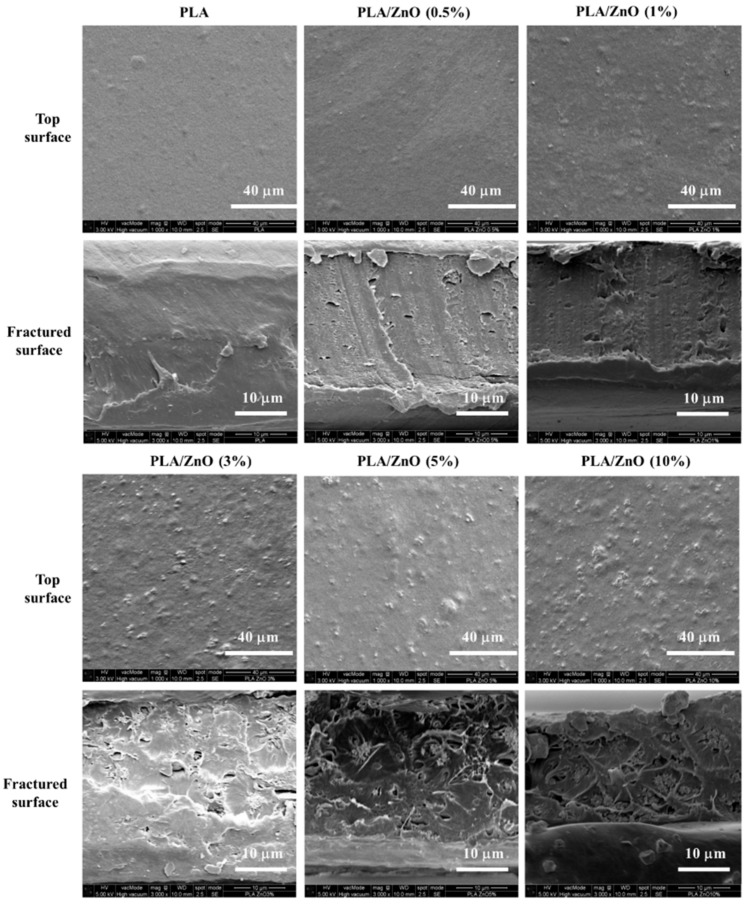
SEM images of the surface and fractured morphology of pure PLA and PLA/ZnO bionanocomposite films.

The SEM images of the top and fractured surfaces of the PLA/ZnO bionanocomposite films are given in Figure 4. A good dispersion of the inorganic filler into the polymer matrix can lead to advanced physical properties in the nanocomposite films [31]. The pure PLA film was smooth at both the top and fractured surfaces. As the ZnO NPs content increased, the top surface of the PLA/ZnO bionanocomposite films showed a higher population of white spots with the existing spots getting rougher. Typically, the Zn–O–Zn bonds are formed between the nano- or micro-sized particles owing to the presence of water molecules, resulting in hard agglomeration, which limit the improvement of the physical properties of the ZnO nanocomposite [32]. Furthermore, a large number of ZnO NPs agglomeration and cavities are observed in the fractured surfaces of bionanocomposite films with over 3% ZnO NPs content. However, it was expected that the positively charged ZnO surface can interact with the unpaired electrons in the carbonyl group of each repeating unit in PLA [23,33]. In addition, it has been reported that ZnO NPs with a homogeneous coral type structure and high surface area can easily interact with the PLA at the interface [34,35]. In contrast, our PLA/ZnO bionanocomposite films had a rough surface, hard agglomeration, and irregular morphology, as compared to pure PLA. This indicated a weak interfacial interaction between ZnO NPs and PLA, as concluded from the FTIR results. 

### 3.5. Thermal Properties 

**Figure 5:**
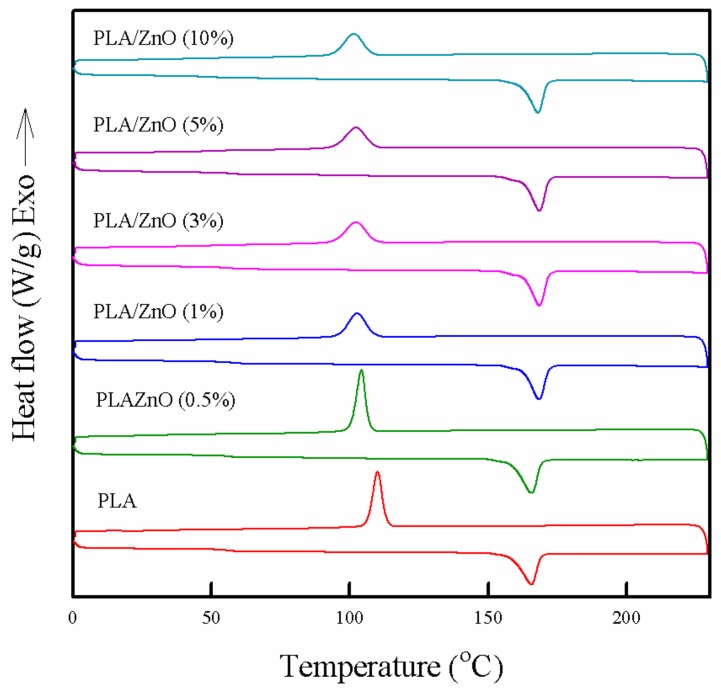
DSC thermograms of pure PLA and PLA/ZnO bionanocomposite films.

The thermal properties of the PLA/ZnO bionanocomposite films were compared with those of the pure PLA film, as shown in Figure 5 and Table 1. The pure PLA film exhibited a melting temperature (*T*_m_) of 165.6 °C and crystallization temperature (*T*_c_) of 110.6 °C, which were related to the solid–liquid and liquid-solid phase transitions, respectively. The melting enthalpy (**Δ***H*_m_) and crystallization enthalpy (**Δ***H*_c_) of the pure PLA film were 46.2 and 43.1 J/g, respectively. As ZnO NPs content increased, a very slight increase in *Tm* was observed, whereas *Tc* was apparently decreased. The slight increase in *Tm* can be explained by the presence of ZnO NPs in the PLA matrix obstructing the chain motion in the crystalline region, which delayed melting of the last crystal in the PLA [36]. Generally, a change in *Tc* depends on the degree of crystallinity [37]. The degree of crystallinity can be estimated by using the heat of fusion in a DSC thermogram [38]. The total **Δ***H*_m_ and **Δ***H*_c_ of the PLA/ZnO bionanocomposite films decreased from 46.2 to 33.9 J/g and from 43.1 to 24.8 J/g, respectively, with increasing ZnO content. It means that the crystallinity of PLA was decreased with introducing ZnO NPs, which is well consist with XRD results. PLA showed high crystallinity with good regularity because of a number of chiral repeating units and correspondingly, the stereoregularity of the polymer [39]. However, the PLA/ZnO bionanocomposite films did not show good dispersion and interaction, which might hinder the regularity and closed packing of PLA polymer chains and induce a decrease in crystallinity of high crystalline PLA [22,40]. This decrease in crystallinity resulted in the apparent decrease in the *T_c_* and *T_g_* values, which is closely related to the segmental mobility and the ordered packing of polymer chains. In general, fillers can enhance the *T*_g_ of polymer films by restricting the mobility of the polymer chains adjacent to the filler surface [12,13]. However, as shown in Figure 5, the *T*_g_ of the pure PLA film was 62.5 °C, whereas the *T_g_* of the PLA/ZnO bionanocomposite films decreased from 62.1 to 58.2 °C as the ZnO content increased. Generally, the electrostatic interaction between the NPs and polymer can increase the crystallinity and *T*_g_ value [41]. Unexpectedly, on increasing the ZnO NPs content, our as-prepared bionanocomposite films showed a decrease in the *T*_g_ value as a result of poor dispersion and the interaction between the ZnO and PLA polymer matrix. 

**Figure 6:**
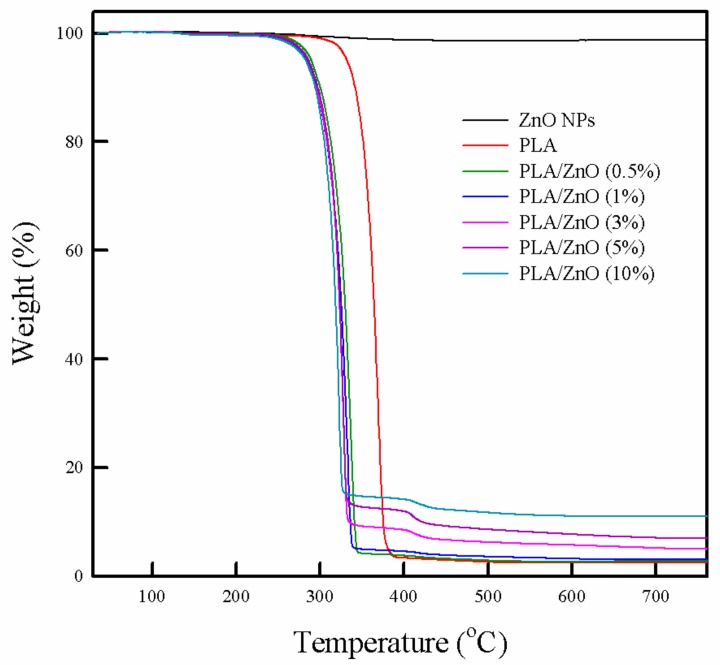
Thermogravimetric analysis of ZnO, pure PLA, and PLA/ZnO bionanocomposite films.

The TGA curves of the PLA/ZnO bionanocomposite films are shown in Figure 6 and the results are summarized in Table 1. All the PLA/ZnO bionanocomposite films showed three stages of weight loss. The first weight loss at 140 °C was due to the loss of moisture content. In the next stage, the pure PLA film showed 96% weight loss in the range of 300 °C to 382 °C, which corresponded to the removal of the ester group on de-polymerization [42,43,44]. The second weight loss (85.3 to 95.7%) of the PLA/ZnO bionanocomposite films occurred in the range of 240 °C to 345 °C, which was a slightly lower temperature as compared to the second weight loss for the pure PLA film. Thereafter, the bionanocomposite films with 3–10% ZnO NPs exhibited 88.7% to 93.5% of weight loss in the range of 400 °C to 460 °C in the third stage, which can be attributed to the thermo-degradation of the polymer chains. It has been previously reported that introducing NPs into the PLA matrix can increase the thermal stability of the polymer films [10,24]. However, in this work, the addition of ZnO NPs into the PLA matrix did not lead to an increase in the thermal stability because of poor dispersion and agglomeration of the ZnO NPs in the PLA polymer matrix. 

It is expected that positively charged surface ZnO NPs tend to interact with the negatively charged oxygen in the unpaired electrons of the PLA polymer due to the electrostatic interaction [33]. However, both the DSC and TGA results indicated that the introduction of ZnO NPs into the PLA matrix did not improve the thermal properties in the composite films because of the weak interfacial interactions and poor dispersion. This result is consistent with several previous reports [24,45].

### 3.6. Barrier Property 

**Figure 7:**
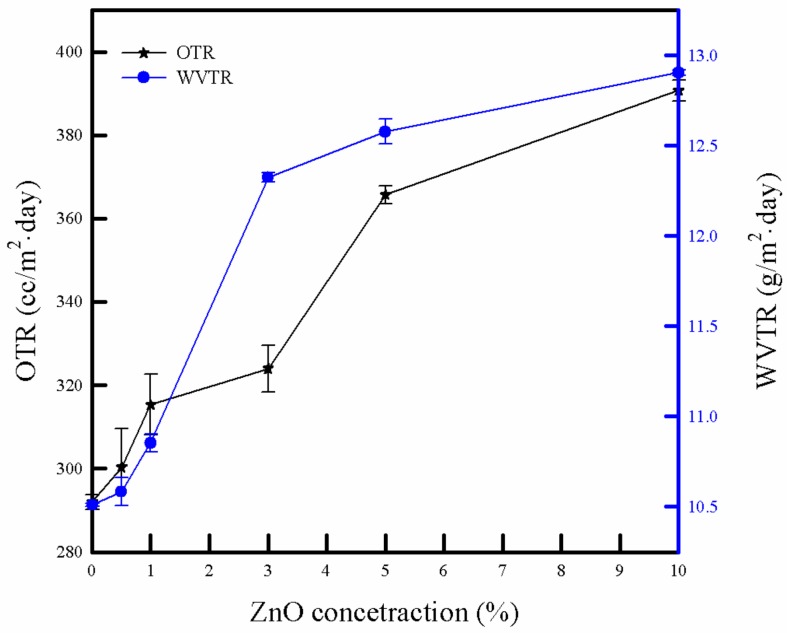
Oxygen transmission rates (OTR) and water-vapor transmission rates (WVTR) of pure PLA and PLA/ZnO bionanocomposite films.

The barrier properties of the packaging material are a key factor in protecting the packaged product. The OTR and WVTR of the PLA/ZnO bionanocomposite films are depicted in Figure 7. The OTR and WVTR values of the pure PLA film were 292 cc/m^2^ day and 10.5 g/m^2^ day, respectively. With the increasing ZnO NPs content, the bionanocomposite films showed an increase in the OTR and WVTR from 300.3 to 390.6 cc/m^2^ day and from 10.6 to 12.9 g/m^2^ day, respectively. Typically, it is expected that the barrier properties of the composites can be improved by incorporating NPs into the polymeric matrix. There are two important factors related to barrier properties, i.e., the chemical and morphological structures. The high chemical interaction, good dispersion, and high crystallinity can enhance the barrier properties of the composite film [46]. In our study, with increasing ZnO NPs content, a decrease in crystallinity was observed in the DSC results. It can be attributed to weak interactions between PLA and ZnO NPs, the cavities in the polymer matrix, as well as poor dispersion and hard agglomeration of the ZnO NPs in the PLA bionanocomposite films. The increasing number of cavities provided pathways in the polymer matrix, which led to imperfectness in the water and oxygen transmission, ultimately resulting in poor barrier properties of the bionanocomposite films [24]. 

### 3.7. Surface Energy and Water Sorption

**Figure 8:**
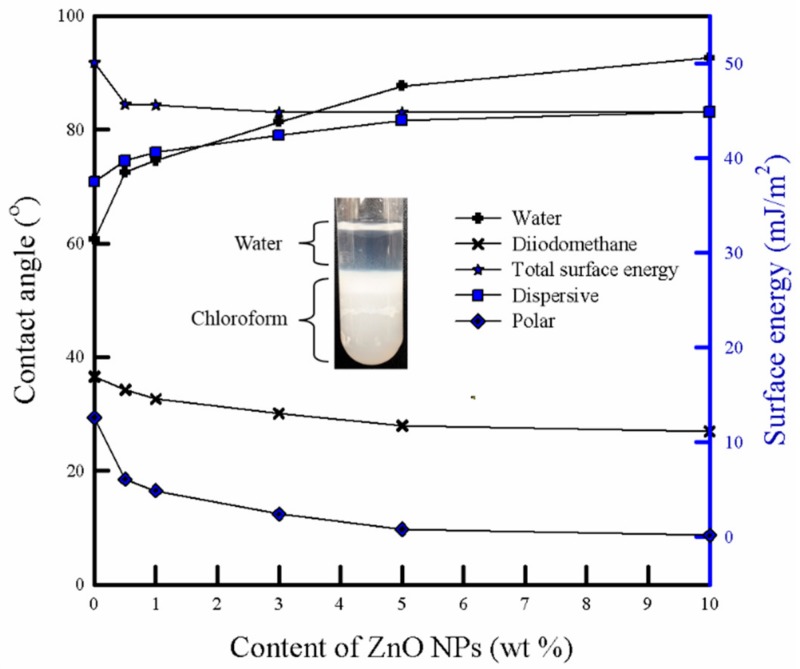
Contact angle and surface energy of pure PLA and PLA/ZnO bionanocomposite films. The photo image shows the dispersion property of synthesized ZnO NPs in a water/chloroform mixture.

Figure 8 depicts the contact angle and surface energy of the PLA/ZnO bionanocomposite films using two liquids, i.e., deionized water and diiodomethane. The pure PLA films showed a water contact angle of 60.7°. With the increasing ZnO content, the water contact angle increased from 72.5° to 92.6°, indicating an increase in the hydrophobicity of the pure PLA film. The total surface energies of the bionanocomposite films were determined by the Owens–Wendt method (geometric mean combining rule), which utilized the contact angle measurement of deionized water and diiodomethane [20]. As ZnO NPs content increased, the bionanocomposite films showed an increase in the surface energy for the dispersive part and decrease in the surface energy for the polar part. Moreover, the bionanocomposite films showed a decrease in the total surface energy. This behavior indicated that the bionanocomposite film with a relatively high ZnO NPs content became more hydrophobic. Mostly, the chemical affinity and surface roughness can affect the contact angle and surface properties of the polymeric composite film. Generally, ZnO NPs have two types of dominant surfaces: Non-polar (1010) and (1120) surfaces, and polar (0001) surface, terminated either by Zn, (0001)-Zn, or O, (0001)-O. Contrary to the non-polar surfaces, the polar surfaces exhibit a significant dipole moment perpendicular to the surface [47]. The (1010) planes are composed of equivalent O^2−^ and Zn^2+^ ions at the same planes, indicating non-polar planes with the lowest surface energy [48]. In our study, the surface of synthesized ZnO NPs can be considered as non-polar because of a relatively better dispersion in chloroform compared to water, as shown in the photo-image of Figure 8 [49]. Further, the SEM results (Figure 4) exhibited that the bionanocomposite film with higher ZnO NPs content showed a rougher surface owing to its agglomeration and weak interaction between the NPs and PLA matrix.

As shown in Table 1, the pure PLA film exhibited a lower water uptake as compared to the PLA/ZnO bionanocomposite films. With the increasing ZnO NPs content, the water uptake increased. The water adsorption of the polymeric materials depends on the chemical affinity, crystallinity, and voids/pore in the materials [20]. As explained for the surface energy aspect, the bionanocomposite film with relatively high ZnO NPs content became more hydrophobic. This property reduced the water uptake in the bionanocomposite films. However, the bionanocomposite films showed a higher number of cavities and a decrease in the crystallinity (higher free volume), i.e., morphological imperfectness, as observed by the SEM and DSC results. The water molecules can easily enter into cavities at the interface between the NPs and the surrounding polymer, and then diffuse into the amorphous region (free volume). As a result, the bionanocomposite films showed a very slight increase in water uptake. 

### 3.8. Antibacterial Activity of PLA/ZnO Bionanocomposite Films

There are numerous attempts aligned with packaging that are being advanced to suppress the bacterial contaminants among foodstuffs. To prepare antibacterial packaging films, the two most common methods are coating antimicrobial agents onto plastic films and incorporating volatile or non-volatile antimicrobial agents into the polymers. The antimicrobial efficiencies of the bionanocomposite films with different contents of ZnO NPs were evaluated according to the JIS Z 2801 standard [24]. The microorganisms used in this study were *S. aureus* and *E. coli*, and the antibacterial activity results are summarized in Table 2. All bionanocomposite films were compared with the pure PLA film as the control sample. 

The efficiency of the antimicrobial activity of the PLA/ZnO bionanocomposite films varied based on the type of microorganism and the amount of loaded ZnO NPs. There were a very high number of microorganisms in the case of the pure PLA film, indicating no antibacterial activity against Gram-positive and Gram-negative bacteria. However, with the increasing ZnO content, the antimicrobial activity (%R) of the bionanocomposite film became very high (%R). Furthermore, the bionanocomposite films demonstrated higher antimicrobial efficiency against *E. coli* as compared to *S. aureus*. 

The antibacterial properties of ZnO may be attributed to hydrogen peroxide (H_2_O_2_) and reactive oxygen species (ROS) (•O^2−^), which inhibit bacterial growth [50,51]. The ZnO NPs discharged Zn^2+^ ions, which passed through the cell wall of the bacteria and reacted with the cytoplasmic content, thereby killing the bacteria. It is known that ZnO NPs produce H_2_O_2_, a strong oxidizing agent, which has the capability of damaging the cell membrane of bacteria [52]. Although the precise antimicrobial mechanism of ZnO has not been disclosed, the antimicrobial efficiency of PLA is attributed to various factors [15], such as the production of ROS (e.g., hydroxyl radicals, hydrogen peroxide, and superoxide) and their photocatalytic activities (activation by visible light and UV), the formation of Zn^2+^ ions, and electrostatic interactions. The ROS enter the microbes and damages the cell membrane, while the Zn^2+^ ions are toxic to the microbes. In our study, the ZnO NPs were prepared by using DDAB, a cationic surfactant, which induced a positive charge on the surface of the NPs. The positive surface charge of ZnO NPs is an essential feature for excellent antibacterial activity. The ZnO NPs with the positive charge (zeta potential of +34 mV) are able to produce electrostatic force to interact with the negatively charged membrane of bacteria, and thus damage the cell membrane [36,52]. It can be reasoned that the antimicrobial activity of ZnO NPs relies on the cell-wall structure of the bacteria. The cell walls of Gram-positive and Gram-negative bacteria are multilayer peptidoglycans with a complex structure. The Gram-negative bacteria, such as *S. aureus*, contain a thicker peptidoglycan layer in the cell wall. On the other hand, the Gram-negative bacteria like *E. coli* have a thin peptidoglycan layer with an additional outer membrane consisting of lipopolysaccharides. This membrane carries a negative charge with high efficiency to the electrostatic interaction with positively charged ZnO NPs. Therefore, the bionanocomposite films showed the stronger antimicrobial activity against *E. coli* than *S. aureus*. 

In previous studies, the production of ROS and deposition of ZnO NPs in the cytoplasm or on the surface of the bacteria led to the reduction in number or death of the bacterial cells. Zang et al. reported that a PLA film incorporating 3% ZnO NPs had ~60.0 and 55.8% R antibacterial efficiencies for *S. aureus* and *E. coli*, respectively [53]. However, in our study, the PLA/ZnO 3% bionanocomposite films showed better bactericidal efficacy against *S. aureus* (83.7% R) and *E. coli* (100% R). Overall, this study indicates that PLA/ZnO bionanocomposite films can be applied as antimicrobial packaging films for food products. However, the improved physical properties at relatively lower content of ZnO NPs can be obtained by enhancing the dispersibility of the ZnO NPs in the PLA matrix.

## 4. Conclusions

This work focused on the preparation of PLA/ZnO bionanocomposite films by the solvent casting method. The physical properties such as optical characteristics, morphology, thermal stability, and barrier properties of the as-prepared PLA/ZnO bionanocomposite films were evaluated depending on the ZnO NPs content. The bionanocomposite films with relatively low ZnO NPs content (0.5 and 1%) showed smooth surfaces and an increase in crystallinity owing to good dispersion and the nucleation effect of the ZnO NPs. In contrast, the bionanocomposite films with relatively high ZnO NPs content (over 3%) exhibited rough surfaces and a decrease in crystallinity because of agglomeration and poor dispersion. Incorporating ZnO NPs into the PLA resulted in a decrease in the thermal stability and barrier properties, but an increase in the hydrophobicity. Moreover, the ZnO/PLA bionanocomposite films exhibited excellent antibacterial activity against Gram-positive (*S. aureus*) and Gram-negative (*E. coli*) bacteria, in addition to acting as a good UV-light barrier. Notably, the bionanocomposite films with over 3% ZnO NPs showed remarkable antibacterial activity against *E. coli*. However, the PLA/ZnO bionanocomposite films need to be further improved to be applicable in food packaging with good barrier and thermal stability, which can be achieved by surface modification of the ZnO NPs with a silane coupling agent.

## Figures and Tables

**Table 1:** Thermal properties of pure PLA and PLA/ZnO bionanocomposite films.

Sample Code	DSC					TGA			DVS
	*T*_g_ (°C) ^a^	*T*_m_ (°C) ^b^	**Δ***H*_m_ (J/g) ^c^	*T*_c_ (°C) ^d^	**Δ***H*_c_ (J/g) ^e^	*T*_d3%_ (°C) ^f^	*T*_d10%_ (°C) ^g^	Residual Content (%) ^h^	Water Uptake (3h)
ZnO NPs	-	-	-	-	-	-	-	98.8	-
PLA	62.5 ± 0.05	165.6 ± 0.3	46.2 ± 1.4	110.6 ± 0.1	43.1 ± 0.2	324.8	341.7	2.6	0.82 ± 0.04
PLA/ZnO (0.5%)	62.1 ± 0.03	166.5 ± 0.3	43.8 ± 0.8	104.5 ± 0.1	35.7 ± 3.1	281.4	300.4	2.8	0.92 ± 0.04
PLA/ZnO (1%)	61.7 ± 0.05	166.6 ± 1.8	40.6 ± 0.1	101.5 ± 2.3	30.9± 0.5	275.7	295.7	3.1	0.94 ± 0.02
PLA/ZnO (3%)	61.3 ± 0.05	166.7 ± 2.4	40.4 ± 1.1	101.1 ± 1.2	30.2 ± 0.5	273.6	294.8	5.2	0.90 ± 0.02
PLA/ZnO (5%)	60.9 ± 0.03	166.8 ± 2.5	38.9 ± 1.8	99.1 ± 4.2	28.7 ± 1.3	273.1	293.7	7.1	0.97 ± 0.01
PLA/ZnO (10%)	58.2 ± 0.07	166.9 ± 3.0	33.9 ± 5.1	98.6 ± 1.8	24.8 ± 4.0	272.6	293.1	11.1	0.94 ± 0.03

^a^ Glass transition temperature of PLA and PLA/ZnO bionanocomposite films. ^b^ Melting temperature of PLA and PLA/ZnO bionanocomposite films. ^c^ Melting enthalpy of PLA and PLA/ZnO bionanocomposite films. ^d^ Crystallization temperature of PLA and PLA/ZnO bionanocomposite films. ^e^ Crystallization enthalpy of PLA and PLA/ZnO bionanocomposite films. ^f^ Temperature at 3 wt % loss of PLA and PLA/ZnO bionanocomposite films. ^g^ Temperature at 10 wt % loss of PLA and PLA/ZnO bionanocomposite films. ^h^ Weight percentage of residues remaining at 700 °C.

**Table 2:** Antibacterial activity of the PLA/ZnO bionanocomposite films.

Sample Code	*S. aureus*	*R* (%)	*E. coli*	*R* (%)
	10^−7^		10^−5^	
PLA	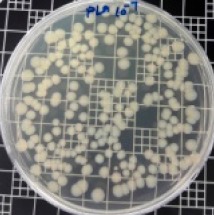	-	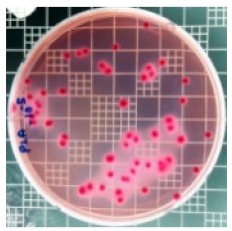	-
PLA/ZnO (0.5%)	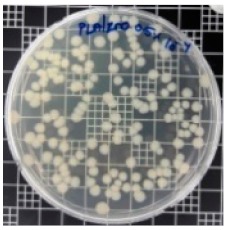	23.3	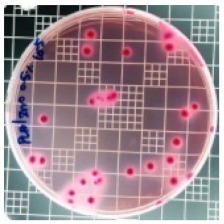	51.7
PLA/ZnO (1%)	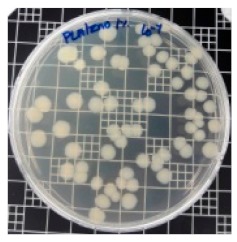	69.2	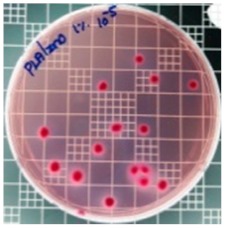	69.0
PLA/ZnO (3%)	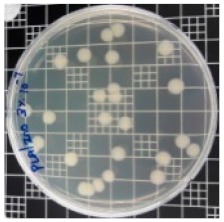	83.7	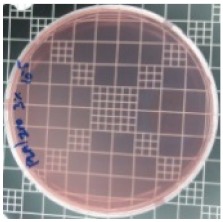	100
PLA/ZnO (5%)	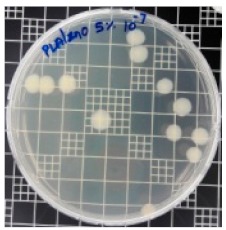	90.2	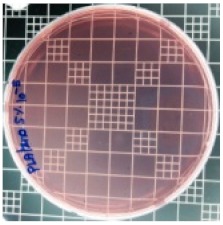	100
PLA/ZnO (10%)	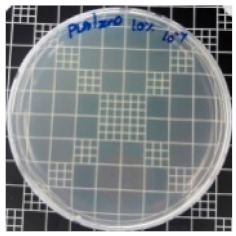	100	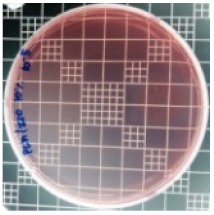	100
